# Psychometric properties of the Audit of Diabetes-Dependent Quality of Life (ADDQoL) in a population-based sample of Polish adults with type 1 and 2 diabetes

**DOI:** 10.1186/s12955-018-0878-y

**Published:** 2018-03-27

**Authors:** Ewelina Bak, Czeslaw Marcisz, Zofia Nowak-Kapusta, Dorota Dobrzyn-Matusiak, Ewa Marcisz, Sylwia Krzeminska

**Affiliations:** 1 Department of Nursing, Faculty of Health Sciences, University of Bielsko-Biala, ul. Willowa 2, 43-309 Bielsko-Biala, Poland; 2 Department of Gerontology and Geriatric Nursing, School of Health Sciences, Medical University of Silesia, ul. Ziolowa 45/47, 40-635 Katowice, Poland; 3 Department of Health Promotion and Community Nursing, School of Health Sciences, Medical University of Silesia, ul. Medykow 12, 40-752 Katowice, Poland; 4 Department of Nursing Propaedeutics, School of Health Sciences, Medical University of Silesia, ul. Francuska 20/24, 40-027 Katowice, Poland; 5 Department of Anxiety Disorders, Hospital of Ministry of Internal Affairs, ul. Glowackiego 10, 40-052 Katowice, Poland; 6 Department of Clinical Nursing, Faculty of Health Sciences, Medical University, ul. Wybrzeze Ludwika Pasteura 1, 50-367 Wroclaw, Poland

**Keywords:** Diabetes mellitus, ADDQoL, Psychometric properties, Internal consistency

## Abstract

**Background:**

The aim of the present paper was the assessment of the psychometric properties of the Polish language version of the Audit of Diabetes-Dependent Quality of Life (ADDQoL) questionnaire applied in Poland among patients with type 1 (T1DM) or type 2 diabetes (T2DM).

**Methods:**

The studies were carried out among 330 patients with diabetes including 115 with T1DM and 215 with T2DM. In all the patients the level of the quality of life was investigated using the Polish language version of the ADDQoL and the psychometric properties were determined taking into consideration internal consistency, the factor loading and intraclass correlations.

**Results:**

It was demonstrated that the values of internal consistency determining the reliability of the Polish language version of the ADDQoL for the overall Cronbach’s alfa coefficient were 0.92 in the studied patients with T1DM and 0.93 in the studied patients with T2DM and the values of the loading factor were respectively 0.39–0.79 and 0.35–0.81. In the study of the correlation between the components of the ADDQoL the correlation coefficients proved to be highly statistically significant: in patients with T1DM *r* = 0.46–0.74 and in patients with T2DM – *r* = 0.42–0.80.

**Conclusion:**

The Polish language version of the ADDQoL is a reliable tool useful for the assessment of the level of the quality of life of adult patients with T1DM or T2DM in Poland and is recommended to be used among Polish-speaking patients with diabetes.

## Background

The treatment procedure in diabetes generally consists in the proper control of the course of this disease with achieving optimum glycaemia results and prevention against the occurrence of complications. For the last twenty years more and more attention has been devoted to the physical, psychical and social aspects of life of persons with diabetes and the embodiment of this trend is these persons’ subjective assessment of the Quality of Life (QoL). Numerous scientific studies have proven that both type 1 diabetes (T1DM) and type 2 diabetes (T2DM) have got a big influence on the level of the quality of life (QoL) of patients. The tools used for assessing the QoL are many acknowledged and widely applied questionnaires which objectify the QoL related to health [1]. These include general questionnaires which assess the QoL such as for example SF-36 [2], WHOQoL [3], as well as questionnaires specific for a given disease which are characterized by high specificity and sensitivity. The specific questionnaires assessing the QoL of patients with diabetes include i.a. Appraisal of Diabetes Scale (ADS), Audit of Diabetes-Dependent Quality of Life (ADDQoL) [4], Diabetes Care Profile (DCP) [5], Diabetes Impact Measurement Scales (DIMS) [6], Diabetes-Specific Quality-of-Life Scale (DSQoLS) [7]. The QoL in diabetes depends on many sociodemographic and clinical factors [8–10]. It has been demonstrated that significant impact on the lowering of the QoL in patients with diabetes is exerted by the complications related to this disease, by the necessity to take insulin and by the co-occurrence of obesity [11]. Also in another paper the patients with diagnosed diabetes related complications reported significantly lower total scores of the DSQoLS [12].

In patients with diabetes the QoL decreases both in the psychical, physical and the social realm which is related to, i.a. the lack of acceptance of the disease. The level of the QoL related to the disease as well as its changes progressing over time should also be an important element of the monitoring of the diabetic patient. The tools applied for the assessment of the components of QoL require reliability obtained through determining the psychometric properties of the scale applied for studies. The psychometric properties include determining the structure of the scale and of its internal consistency, validity and reliability. This is achieved thanks to statistical methods such as determining the Cronbach’s alpha, factor loading, floor and ceiling effect, intraclass correlation and factor analysis. An optimal confirmation of the reliability of a scale is obtaining satisfactory psychometric parameters of the applied tool. The questionnaire which has been mainly applied for studying the level of the QoL of patients with diabetes is the ADDQoL, after prior documenting of this scale’s reliability in language versions appropriate for the studied populations, i.a. in Slovenian [13], Portuguese [14], Greek [15], Norwegian [16], Chinese [17–19], Malay [20]. It was demonstrated that the ADDQoL had good psychometric properties and provided clinicians and researchers with a useful tool for the comprehensive assessment of the QoL in adults with diabetes [11, 18]. This questionnaire constitutes a tool allowing for individualizing the measurement of the QoL of patients with diabetes with taking into consideration 19 domains of this quality [4]. This allows the respondent to indicate the life domains which are not applicable or applicable in terms of the significance of the influence of diabetes on particular components of the QoL. Considering the fact that the studied populations of patients with diabetes differ in particular countries in respect of sociodemographic issues, ethnic issues, culture, wealth, lifestyle and the provided medical care, determining the QoL of these patients requires analyzing the psychometric properties of the questionnaires applied in the mother tongue of the assessed population. The psychometric properties of the Polish language version of the ADDQoL applied among the native inhabitants of Poland suffering from diabetes have not been determined until now.

The assessment of the reliability of the Polish language version of the ADDQoL applied in Poland in patients with T1DM or T2DM was the aim of the present study.

## Methods

### Participants

All the ill studied subjects were patients of the Regional Hospital in Bielsko-Biala (the Diabetic Clinic), the Diabetic Unit of the Medi-Diab Non-Public Medical Center and the Diabetic Unit in Katowice. Diagnosing T1DM or T2DM was performed and documented by physicians specialized in diabetology. The survey studies were carried out by the authors of the paper (EB, DDM) among subsequent patients of outpatient clinics in the period from March 2016 till January 2017. All the studied patients were native inhabitants of the Southern regions of Poland.

The inclusion criteria to the groups of studied patients were as follows: age from 18 to 60, the occurrence of diabetes lasting for at least a year and the ability to complete a questionnaire written in Polish. The persons excluded from the study were patients with secondary diabetes, gestational diabetes, patients in whom acute inflammation requiring treatment occurred during the last 3 months, patients taking immunosuppressive drugs, glucocorticoids, anti-inflammatory drugs, sedative and psychoactive drugs as well as patients with diagnosed cancer, with function disorders of the thyroid and the adrenal glands, with alcoholism and patients who did not provide consent for performing the study. During the patient history taking (interview) and on the basis of the medical case record (medical history) the authors of the study took account of chronic comorbidities such as the coronary artery disease, heart failure, hypertension, renal failure and stroke as well as late diabetes complications such as retinopathy, nephropathy, polyneuropathy and the diabetic foot. Comorbidities and diabetes complications were diagnosed by specialist physicians, mainly internists, cardiologists, neurologists, nephrologists, diabetologists and ophthalmologists during hospitalization or in specialist clinics.

The treatment of diabetes generally consisted in adhering to a diet and taking insulin in patients with T1DM and a diet, taking derivatives of sulfonylurea and biguanides and in some cases insulin in patients with T2DM.

The study was performed among 330 patients with T1DM and T2DM. Considering the fact that in Poland 9% of the population suffer from diabetes [21] with the assumption that the maximum error is 3% and the confidence level is 90%, the minimum sample size is estimated to be 245 persons. Thus the size of the investigated sample was sufficient for the psychometric analysis. The group of patients with T1DM comprised 115 persons including 57 women and 58 men. The group of patients with T2DM comprised 215 persons including 114 women and 101 men. The patients with T1DM were selected out of 111 randomly chosen women and 115 randomly chosen men with T1DM while the patients with T2DM were selected out of 151 randomly chosen women and 145 randomly chosen men with T2DM. The reasons for reducing the number of patients qualified for further studies were a lack of consent (23 women and 25 men with T1DM, 19 women and 21 men with T2DM), incompletely filled surveys (25 women and 27 men with T1DM, 12 women and 16 men with T2DM); this reduction also resulted from taking into consideration the research inclusion and exclusion criteria: in patients with T1DM among women – hyperthyroidism (1 person), taking sedative or psychotropic drugs (3) and glucocorticoids (2), in men – alcoholism (3 persons), neoplasm (1), glucocorticoids (1), in patients with T2DM among women – hyperthyroidism (1 person), hypothyroidism (1), neoplasm (1), taking sedative or psychotropic drugs (2 persons) and alcoholism (1), in men – alcoholism (3), neoplasm (2), taking immunosuppressive drugs (2).

The research was performed with the consent of the Bioethics Committee of the Beskidzka Regional Chamber of Physicians in Bielsko-Biala; the consent was provided during the meeting held on 11th February 2016 (No. of consent 2016/02/11/1).

### Methods

The values of the glucose measures (fasting glycemia, glycemia 2 h after a meal and the concentration of glycated hemoglobin – HbA1c) and lipid measures (the concentration of LDL-, HDL-, total cholesterol and triglycerides) were determined in all the studied persons. The systolic blood pressure and diastolic blood pressure were measured.

Next, questionnaire studies were carried out using the following questionnaires: the patients’ demographic and clinical data survey and the ADDQoL. The questionnaire was accompanied by brief information about the studied person including parameters such as: age, sex, place of residence, education, marital status, professional activity, body mass, height, used stimulants, comorbidities, the duration of diabetes, the occurrence of diabetes complications and the taken drugs. The ADDQoL questionnaire developed by Clare Bradley version 19 is a tool specific for diabetes which is used for examining the QoL both in T1DM and T2DM [4]. It consists of two general questions referring to the QoL; the first question determines the measurement of the general, present level of the QoL – it includes a 7-grade scale (excellent, very good, good, neither good nor bad, bad, very bad and extremely bad) and the second question determines the concrete influence of diabetes on the QoL – it includes a 5-grade scale (very much better, much better, a little better, the same and worse). The remaining components referred to the 19 domains of the QoL without the disease and the influence of diabetes on the aspects of health. Each domain includes two components: Impact (from − 3, maximum negative impact of diabetes, to + 1, positive impact of diabetes) and Importance (from 3, very important, to 0, not at all important). The product of impact and importance ratings determines the value of the weighted impact score. This value may range from − 9 to + 3 for every examined domain of the ADDQoL. The lower the value of the weighted impact score the worse the aspect of health or life within the scope of a given domain. The average value of the weighted impact score was also calculated for the whole scale. The ADDQoL comprises the following domains: leisure activities, working life, journeys, holidays, physical health, family life, friendship and social life, personal relationship, sex life, physical appearance, self-confidence, motivation, people’s reactions, feelings about future, financial situation, living conditions, dependence on others, freedom to eat and freedom to drink.

The ADDQoL was applied in the studies with the consent and license received from Clare Bradley from the Health Psychology Research, Department of Psychology, Royal Holloway, University of London. The license of the Polish language version was marked with the number CB521.

Before commencing the study every person was informed about its purpose. The questionnaire was filled in personally and anonymously by the patients during the physician’s visit. The time needed for filling in the survey was 15–20 min.

### Statistical analysis

The statistical analysis was performed using the Statistica v.10 StatSoft Poland software. The level of statistical significance assumed in all the calculations was α = 0.05. Basic statistics, i.e. the mean and the standard deviation were calculated for quantitative data. The Mann–Whitney U test was used in order to compare two independent groups characterized by distributions which were not normal. The Chi-square test or the Yates-corrected Chi-square test were used for checking the occurrence of relationships between the considered variables. The analysis of the correlation between the weighted impact scores obtained from particular domains and the general ADDQoL result, which was the mean from all the 19 domains considered, was performed basing on Spearman’s correlation coefficient. The reliability of the applied questionnaire was checked by calculating the Cronbach’s alpha coefficient (> 0.7 was considered acceptable) for the ADDQoL. The factor structure was explored using the Principal Components Analysis. A forced one-factor solution was obtained to confirm the validity of calculating the ADDQoL score. Variables with loadings lower than 0.4 were considered to load unsatisfactorily. Ceiling or floor effects were considered to be present if more than 15% of respondents reported the highest or lowest possible score, respectively. The validity of the measurement of the ADDQoL survey was verified with the help of the factor analysis using the maximum likelihood method because this method allows for verifying the model’s goodness of fit. The number of patients in both studied groups was over 100 thus the requirement for applying the factor analysis was met. Bartlett’s Test of Sphericity was applied in order to verify the relevance of applying the factor analysis whereas in order to assess the adequacy of the correlation matrix the Kaiser-Mayer-Olkin (KMO) coefficients were determined. A value exceeding 0.7 indicates the relevance of performing the factor analysis and Bartlett’s Test of Sphericity verifies the hypothesis on the unitary correlation matrix. If it is significant this means that the factor model is appropriate for the analysed variables. The model’s goodness of fit to the data was calculated using the U test. This test verifies whether all the residual correlations are equal 0, i.e. whether the residual correlation matrix is a diagonal matrix.

## Results

### General characteristics of the study groups

The sociodemographic, clinical and biochemical characteristics of the studied patients have been presented in Table 1. The age of the patients with T1DM was 29.8 ± 8.2 (mean ± SD) and the age of the patients with T2DM was 52.2 ± 8.5. In terms of education the studied persons were divided into those with primary education and additionally with 3-year vocational education, with secondary education without a diploma, with secondary education with graduation, with university higher education or vocational higher education. The comorbidities did not include clinically symptomatic peripheral arterial dieases neither any history of myocardial infarcts, strokes.

**Table 1:** Sociodemographic, clinical and biochemical characteristics of the studied patients (mean ± standard deviation)

Parameter		Type 1 diabetes (*n* = 115)	Type 2 diabetes (*n* = 215)
Age (years)		29.8 ± 8.2	52.2 ± 8.5
Body mass index (kg/m^2^)		24.9 ± 3.8	29.4 ± 5.5
Education	Primary and Vocational/Preuniversity/Higher	30/42/43	122/70/23
Place of residence	Rural/Urban	38/77	164/51
Marital status	Single/Married/Widower/Divorced	32/74/2/7	14/192/2/7
Professional activity	Currently working/Not working	91/24	161/54
Smoking	Never/Past/Present	42/34/39	95/65/55
Alcohol	Drinking/Not drinking	67/47	77/137
Systolic blood pressure (mmHg)		125.1 ± 11.5	132.0 ± 16.4
Diastolic blood pressure (mmHg)		78.8 ± 10.2	82.3 ± 12.3
Duration of diabetes (years)		9.3 ± 7.2	9.4 ± 7.1
Comorbidities	Coronary artery disease	1	55
	Hypertension	13	136
	Heart failure	1	33
	Renal failure	1	26
Drugs	Oral antidiabetic/insulin/antihipertensive/statins	0/115/13/0	215/100/144/97
Complications of diabetes	Visual disturbances/nephropathy/polyneuropathy/diabetic foot	20/0/7/1	60/21/35/8
HbA1c (%)		6.03 ± 0.99	6.66 ± 1.29
Glucose fasting (mg/dl)		120.9 ± 24.3	129.5 ± 30.5
Glucose post-meal (120 min) (mg/dl)		137.9 ± 24.4	149.9 ± 33.3
Total cholesterol (mg/dl)		158.3 ± 26.9	185.1 ± 37.3
LDL-cholesterol [mg/dL]		95.3 ± 29.3	101.5 ± 29.8
HDL-cholesterol [mg/dL]		58.5 ± 14.6	54.4 ± 35.0
Triglycerides [mg/dL]		135.5 ± 24.1	148.1 ± 45.8

*HbA1c* glycated hemoglobin, *LDL* low density lipoprotein, *HDL* high density lipoprotein

Table 2 presents the diabetic patients’ answers to the following questions: “In general, my present QoL is” and “If I did not have diabetes, my QoL would be?”. There were 7 possible answers to the first question: excellent (+ 3 points), very good (+ 2), good (+ 1), neither good nor bad (0), bad (− 1), very bad (− 2), extremely bad (− 3). There were 5 possible answers to the second question: very much better (− 3 points), much better (− 2), a little better (− 1), the same (0), worse (1).

**Table 2:** The general quality of life of women and men with diabetes

	Type 1 diabetes (*n* = 115)	Type 2 diabetes (*n* = 215)
In general, my present quality of life is:	n(%)	
Excellent (+ 3)	3(2.6)	1(0.5)
Very good (+ 2)	30(26.1)	28(13.0)
Good (+ 1)	48(41.7)	98(45.6)
Neither good nor bad (0)	31(27.0)	63(29.3)
Bad (−1)	3(2.6)	22(10.2)
Very bad (− 2)	0(0.0)	2(0.9)
Extremely bad (−3)	0(0.0)	1(0.5)
mean ± standard deviation	0.99 ± 0.86	0.60 ± 0.92
If I did not have diabetes, my QoL would be:	n(%)	
Very much better (−3)	12(10.4)	23(10.7)
Much better (−2)	30(26.1)	70(32.6)
A little better (−1)	52(45.2)	82(38.1)
The same (0)	21(18.3)	39(18.1)
Worse (1)	0(0.0)	1(0.5)
mean ± standard deviation	−1.29 ± 0.89	− 1.35 ± 0.91

The frequencies of using the NA (not applicable) responses, both in impact and importance rating were higher among patients with T2DM than with T1DM: “Working life” (36.28 and 9.52, 10.48, respectively), “Holidays” (7.91, 8.84 and 4.76), “Personal relationship” (6.05, 6.51 and 4.76, 2.86) (Table 3). For some domains the respondents assigned zero value to the importance rating (Table 3), which indicated that certain domains of the QoL assessed by the ADDQoL were not of sufficient importance to them.

**Table 3:** Frequency of applying the ‘NA’ response in impact and importance ratings of the ADDQoL for patients with diabetes

Domains	Type 1 diabetes (*n* = 115)			Type 2 diabetes (*n* = 215)		
	Impact rating	Importance rating		Impact rating	Importance rating	
	NA response [n(%)]	NA response [n(%)]	0 value of importance (% of respondents)	NA response [n(%)]	NA response [n(%)]	0 value of importance (% of respondents)
Leisure activities	0(0)	0(0)	1.36	1(0.47)	5(2.33)	1.01
Working life	10(9.52)	11(10.48)	0.91	78(36.28)	78(36.28)	1.01
Journeys	0(0)	0(0)	10.45	1(0.47)	1(0.47)	3.27
Holidays	5(4.76)	5(4.76)	1.82	17(7.91)	19(8.84)	0.5
Physical health	1(0.95)	1(0.95)	0.91	1(0.47)	2(0.93)	0.75
Family life	0(0)	0(0)	0	1(0.47)	0(0)	0
Friendship and social life	1(0.95)	1(0.95)	0.45	1(0.47)	2(0.93)	0.5
Personal relationship	5(4.76)	3(2.86)	0	13(6.05)	14(6.51)	0.25
Sex life	1(0.95)	1(0.95)	0	18(8.37)	19(8.84)	2.01
Physical appearance	0(0)	0(0)	0.45	4(1.86)	1(0.47)	1.01
Self-confidence	0(0)	0(0)	0.91	0(0)	1(0.47)	0.75
Motivation	1(0.95)	1(0.95)	0	1(0.47)	3(1,4)	0.75
People’s reactions	1(0.95)	1(0.95)	0.91	13(6.05)	2(0,93)	1.76
Feelings about future	1(0.95)	1(0.95)	1.36	1(0.47)	2(0.93)	0.75
Financial situation	1(0.95)	2(1.9)	0.91	1(0.47)	1(0.47)	0.25
Living conditions	1(0.95)	1(0.95)	1.82	2(0.93)	1(0.47)	0.75
Dependence on others	0(0)	0(0)	0	5(2.33)	3(1.4)	1.01
Freedom to eat	0(0)	0(0)	3.64	1(0.47)	2(0.93)	1.26
Freedom to drink	0(0)	1(0.95)	4.09	1(0.47)	2(0.93)	1.26

The values of KMO coefficients in patients with T1DM and patients with T1DM were high, 0.86 and 0.94 respectively, and the statistical significance in Bartlett’s Test of Sphericity was *p* < 0.0001 in both groups which confirmed the relevance of applying the factor analysis. With the help of this analysis in the maximum likelihood method 8 factors (which explain 53.68% of the total variance) were obtained for patients with T1DM and 4 factors (which explain 42.53% of the total variance) were obtained for patients with T2DM. Considering this model representative requires obtaining ≥80% of total variance. On the basis of the factor analysis it was demonstrated that in T1DM the value χ^2^ = 791.71, df = 520, *p* < 0.0001 and in T2DM χ^2^ = 1144.06, df = 662, *p* < 0.0001. The value *p* < 0.05 does not ensure that the residual correlation matrix is significantly different from the diagonal matrix, which means that the calculated model cannot be accepted. This confirms the assumptions of the authors of the ADDQoL survey that all the questions have been formulated correctly, it is not possible to create groups of questions (domains), every question carries important information and it is not possible to reduce the number of the questions without a significant loss of information provided by them.

A forced one-factor Principal Components Analysis indicated that 18 items had factor loadings exceeding 0.4 (Table 4). Only the items: “Dependence on others” in T1DM and “Freedom to drink” in T2DM loaded < 0.4 into this factor, respectively 0.392 and 0.346. The forced one factor solution explained 43.5% of the variance in T1DM and 44.3% of the variance in T2DM.

**Table 4:** The values of α, factor loadings, floor, ceiling effects of weighted impact score for items of ADDQoL for patients with diabetes

Domains	Type 1 diabetes (*n* = 115)				Type 2 diabetes (*n* = 215)			
	α	FL	FE	CE	α	FL	FE	CE
Cronbach’s α	0.92	–	–	–	0.93	–	–	–
Cronbach’s α with deleted item:	0.92	–	–	–	0.93	–	–	–
Leisure activities	0.91	0.766	3.48	0.87	0.92	0.701	5.58	0
Working life	0.91	0.753	4.35	0	0.92	0.779	5.58	0
Journeys	0.91	0.772	4.35	0	0.92	0.736	4.65	0
Holidays	0.91	0.759	4.35	0	0.92	0.807	5.58	0
Physical health	0.92	0.703	8.70	0	0.92	0.719	5.58	0
Family life	0.92	0.704	4.35	0	0.92	0.765	4.19	0
Friendship and social life	0.92	0.693	3.48	0	0.92	0.652	2.33	0
Personal relationship	0.92	0.714	6.09	0	0.92	0.728	5.58	0
Sex life	0.92	0.512	5.22	0	0.92	0.713	6.98	0.47
Physical appearance	0.92	0.538	5.22	0	0.92	0.695	7.91	0
Self-confidence	0.92	0.723	6.09	0	0.92	0.801	6.51	0
Motivation	0.91	0.790	3.48	0	0.92	0.708	5.58	0
People’s reactions	0.92	0.646	2.61	0	0.92	0.656	1.86	0
Feelings about future	0.92	0.636	9.57	0	0.92	0.520	8.37	0
Financial situation	0.92	0.720	3.48	0	0.92	0.595	2.33	0
Living conditions	0.92	0.554	1.74	0	0.93	0.487	2.79	0
Dependence on others	0.92	0.392	8.70	0.87	0.92	0.584	6.05	0
Freedom to eat	0.92	0.490	9.57	0	0.93	0.418	10.23	0
Freedom to drink	0.92	0.477	10.43	0	0.93	0.346	8.37	0
% variance	–	43.5%	–	–	–	44.3%	–	–

*α* Cronbach’s α, *FL* factor loading, *FE* floor effect (%), *CE* ceiling effect (%)

The internal consistency estimate for the 19 items was Cronbach’s α equal 0.92 for T1DM and 0.93 for T2DM (Table 4). When the item with the factor loading < 0.4 was removed, the internal consistency remained at the same level (T1DM: Cronbach’s α = 0.92 and T2DM: Cronbach’s α = 0.93).

In patients with T1DM the percentual values of the floor effect were highest for the following domains: “Physical health”, “Feelings about future”, “Dependence on others”, “Freedom to eat” and “Freedom to drink” and in patients with T2DM: “Feelings about future”, “Freedom to eat” and “Freedom to drink”. The percentual values of the ceiling effect were equal zero for almost all domains, irrespectively of the type of diabetes of the examined persons (Table 4). Values of the floor and ceiling effects were lower than 15% for each of the 19 domains.

The intraclass correlations were determined by calculating Spearman’s correlation coefficient (r). Every item positively correlated with the overall scale in both examined groups with diabetes: in the group of patients with T1DM *r* = 0.46–0.74 (*p* < 0.0001) and in the group of patients with T2DM – *r* = 0.42–0.80 (*p* < 0.0001) (Table 5).

**Table 5:** The correlation coefficients of intraclass correlations of ADDQoL for patients with diabetes

Domains	Overall weighted impact score of ADDQoL	
	Type 1 diabetes (*n* = 115)	Type 2 diabetes (*n* = 215)
Leisure activities	0.61	0.57
Working life	0.63	0.76
Journeys	0.67	0.67
Holidays	0.71	0.71
Physical health	0.71	0.69
Family life	0.61s	0.69
Friendship and social life	0.67	0.69
Personal relationship	0.67	0.69
Sex life	0.65	0.70
Physical appearance	0.59	0.73
Self-confidence	0.69	0.80
Motivation	0.74	0.74
People’s reactions	0.60	0.64
Feelings about future	0.63	0.62
Financial situation	0.67	0.60
Living conditions	0.46	0.42
Dependence on others	0.46	0.63
Freedom to eat	0.49	0.55
Freedom to drink	0.49	0.45

## Discussion

The assessment of the psychometric properties and applicability of the Polish language version of the ADDQoL among patients with diabetes in Poland hasn’t been performed yet. The results obtained in the present paper prove that in terms of feasibility and reliability the Polish language version of the ADDQoL constitutes an appropriate tool for studying the QoL in patients with T1DM or T2DM in Poland. In the applied methodology of assessment of the psychometric properties of the utilized scale, the authors of the present paper took into consideration commonly applied studies: the Cronbach’s alpha indicator determining the internal consistency, factor loadings, intraclass correlation coefficients, the floor and ceiling effects and factor analysis.

The internal consistency estimate for the 19 items indicated very good results, close to the reported value of the original version of the ADDQoL developed by Badley et al. [4]. In case of the modified ADDQoL the internal consistency was similar to that of its English version and the single factor solution was supported [4]. The results mentioned in this paper demonstrate that although the ADDQoL is a sophisticated scale, it may be used in population-based large-scale studies. We have also demonstrated that the values of the loading factor were satisfactory in 18 domains (without “Dependence on others” in T1DM and “Freedom to drink” in T2DM) of the QoL and the intraclass correlation coefficients of the ADDQoL proved to be significant for all the domains of the QoL of patients with diabetes. Considering the above results of our studies and additionally the achieved values of the floor and ceiling effects it is possible to suggest satisfactory reliability of this scale. The appropriate test structure of the ADDQoL applied by us is indicated by the obtained results of the factor analysis, which constitutes good construct validity.

Studies related to the QoL of patients with diabetes carried out using the ADDQoL in various language versions have been conducted in European countries, in Australia, in the United States of America, in Argentina and in Asia. High reliability of the ADDQoL was demonstrated in patients with T2DM in Slovenia [13], in Australia [11], in Argentina [22], in the northeastern part of the United States among Spanish-speaking Puerto Ricans [23]. In Greece the assessment of the QoL was performed in patients with T2DM using the ADDQoL, however the reliability of the scale was not assessed. Even though these studies were not supported by the assessment of validity, they confirmed that the ADDQoL is an important and useful tool aimed at determining the QoL in the period of taking care of patients with diabetes [15]. In Portugal the studies were carried out among people with T1DM and T2DM [14]. It was demonstrated that the Portuguese language version of the ADDQoL had similar psychometric properties as in our own studies. In Norway in turn, in patients with T1DM and T2DM the QoL was studied using the Norwegian version of the ADDQoL, showing the high reliability of this scale and the loading factor values proved to be higher than 0.4 in all domains except for one (“Freedom to drink”) [16], similarly as in our own studies.

If we move to regions which are culturally different from those of Europe, i.e. to the areas of Asia (China, Taiwan, Singapore) we can also observe that the ADDQoL obtained very high validity which proved the consistency of the domains it comprises. Applying the Chinese version of the ADDQoL in studies of the psychometric properties among patients with T2DM showed high reliability with excellent internal consistency of this scale, the factor loadings were > 0.4 for all the items except for “Freedom to drink” [18]. Also in our own study in patients with T2DM the value of the loading factor proved to be lower than 0.4 only for the item “Freedom to drink”.

In our study it was found that the values of the Cronbach’s alpha indicator for all the assessed domains of the scale were comparable in the analyzed groups despite of the differentiation in terms of some of the demographic parameters (age, BMI) and the occurrence of comorbidities and complications of diabetes and the taken medications.

In conclusion it may be stated that the reliability and validity of the Polish language version of the ADDQoL applied in Poland among people with T1DM and T2DM was high, which has been proven by the obtained values of the Cronbach’s alpha, the factor loading and the calculated intraclass correlation coefficients and construct validity of the studied domains within the scale.

This study is associated with some limitations. Firstly, the generalizability of our findings to the general population with diabetes may be limited because the studied population was not too big, especially the number of patients with T1DM which was about 2 times smaller than the number of persons in the group of patients with T2DM. Nevertheless, it may be assumed that the population of patients with T1DM and T2DM examined by us was representative for the patients with diabetes in Poland, especially in terms of the method of treatment and the lifestyle. It should be however noted that despite of the fact that the patients studied by us were selected for the studies randomly, they demonstrated a well controlled course of diabetes which is proven by the concentration of glycated hemoglobin equal on average 6.03% in T1DM and 6.66% in T2DM; perhaps this is due to the fact that these patients were regularly controlled by diabetologists in diabetes treatment centers. Secondly, no other scales determining the QoL were used in order to make a comparison with the results of the assessed psychometric properties of the ADDQoL. Despite of these limitations, our findings might provide important information regarding the determinants of the QoL in patients with T1DM and T2DM in Poland.

## Conclusion

The ADDQoL in the Polish language version is a reliable tool which may be applied for the assessment of the individual quality of life of patients with type 1 and type 2 diabetes in Poland and is recommended to be used among Polish-speaking patients with diabetes.

## Abbreviations

ADDQoL: the Audit of Diabetes-Dependent Quality of Life
ADS: Appraisal of Diabetes Scale
BMI: Body Mass Index
CE: ceiling effect
DCP: Diabetes Care Profile
DIMS: Diabetes Impact Measurement Scales
DSQoLS: Diabetes-Specific Quality-of-Life Scale
FE: floor effect
FL: factor loading
KMO: Kaiser-Mayer-Olkin
NA: not applicable
r: Spearman’s correlation coefficient
T1DM: patients with type 1 diabetes
T2DM: patients with type 2 diabetes
